# Nonextraction Management of Severely Malaligned and Constricted Upper Arch

**DOI:** 10.1155/2020/8836061

**Published:** 2020-08-25

**Authors:** Manar K. Hajrasi, Ahmad A. Al-Fraidi, Abdulkarim A. Hatrom, Ali H. Hassan

**Affiliations:** ^1^Orthodontic Division, Jeddah Specialty Dental Center, Ministry of Health, Jeddah, Saudi Arabia; ^2^Orthodontic Division, Specialized Dental Center, King Fahad Hospital, Madina, Saudi Arabia; ^3^Orthodontic Division, Al Noor Hospital, Ministry of Health, P.O. Box 4137, Makkah 24359, Saudi Arabia; ^4^Department of Orthodontics, Faculty of Dentistry, King Abdulaziz University, P.O. Box 80209, Jeddah 21589, Saudi Arabia; ^5^Al-Farabi Private Colleges, P.O. Box 23643, Jeddah 21589, Saudi Arabia

## Abstract

This case report presents the treatment of a 12-year-old female with a severely crowded upper arch, severely palatally displaced upper premolars and lateral incisors, large midline diastema, lower midline deviation to the right, class III dental and skeletal relationships due to mild maxillary deficiency, retroclined lower incisors, straight profile, and retrusive lips. A nonextraction treatment approach is described, in which the upper and lower arches were expanded to their original three dimensions using a trihelix expander, a lip bumper appliance, and a fixed orthodontic appliance. Retention was also planned in accordance with the original malocclusion, which inclued a full-time-wear upper wraparound retainer, upper and lower anterior fixed lingual retainers, upper frenectomy, and fibrotomy for rotated teeth. *Conclusion*. Severe malalignment of teeth does not necessarily require extraction treatment. Gaining space is an art that requires a proper assessment of the anteroposterior and transverse dimensions of alveolar arches, lip prominence, and postorthodontic stability.

## 1. Introduction

Crowding and malalignment of teeth are among the common malocclusions seen in orthodontic practice [1, 2]. Treatment strategies usually involve either extraction or expansion approaches, depending on several factors, such as the amount of crowding, lip prominence, and transverse dimensions of the dental arches [3, 4]. There is considerable controversy in the literature about whether long-term results are better achieved by extraction or nonextraction therapy, especially in borderline cases. Those who are in favor of nonextraction therapy often claim that extraction treatment tends to dish lips, while those who are in favor of extraction often presume that the lips tend to protrude as a result of excessive incisor flaring, which may result in excessive lip strain and lip incompetence [1, 5]. Previous studies reported that patients who had extraction orthodontic treatment tend to end up with lips that are 2 to 4 mm flatter, on average, than nonextraction patients at the end of treatment [5]. Other studies reported no difference between extraction patients and nonextraction patients as perceived by lay people [6]. In addition, several reports have indicated that the increases in dental arch length and width during orthodontic treatment tend to relapse to pretreatment values after both extraction and nonextraction therapy [7, 8], especially mandibular intercanine and intermolar width dimensions [7, 9]. The maintenance of the pretreatment values for intercanine and intermolar distances was suggested as the key to posttreatment stability because these values were believed to represent a position of muscular balance for patients [7–9].

Nevertheless, the decision to either extract or not extract is challenging. Orthodontists might be misled in cases with severe malalignment if they do not carefully evaluate the space available in three dimensions, as well as the lip position.

In this report, the nonextraction approach of orthodontic treatment of a patient with severely malaligned upper teeth is described, in which the upper and lower arches were expanded to their original three dimensions using a trihelix expander, a lip bumper appliance, and a fixed orthodontic appliance, paying attention to the lip position, transverse dimensions of the dental arches, space available, and the long-term stability retention.

## 2. Diagnosis and Etiology

A 12-year-old female patient, in the late mixed dentition stage, presented with a chief complaint of crooked teeth. Medical and dental histories were insignificant.

**Figure 1:**
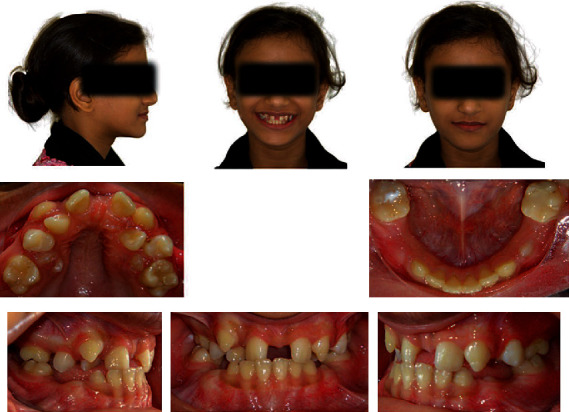
Pretreatment extraoral and intraoral photographs.

Extraoral examination revealed the following: anteroposteriorly, the patient had a straight profile, average nose and chin, retrusive lips, average nasolabial angle, 90-degree cervicomental angle, and normal labiodental fold. She had an average mandibular plane angle and an average lower anterior face height. Frontally, the patient had a mesofacial, fairly symmetrical face with a flat arc of the smile. Temporomandibular joints were within normal limits (Figure 1).

Intraoral examination revealed generalized marginal gingivitis with adequate attached gingivae, physiological gingival pigmentation on the labial of the lower anterior gingiva, healthy oral mucosa and tongue, occlusal filling on lower first molars, unerupted lower right premolars, and lower left second premolar and mild lateral shift of the mandible to the right (Figure 1).

**Figure 2:**
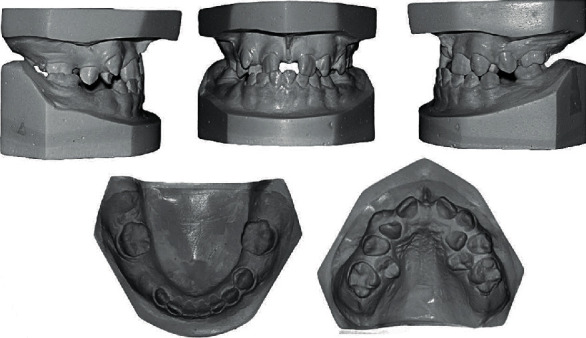
Pretreatment study models.

The model analysis showed class I molar and buccal segment relationships with a class III incisor relationship, and shallow overbite and overjet. The upper arch was “*Ω*” in shape and constricted. The upper midline was indeterminate due to the presence of large diastema (7 mm); the lower midline was deviated to the right by 1.5 mm relative to the philtrum; teeth #12, 14, 15, 16, 22, and 25 were in crossbite; the upper lateral incisors and upper second premolars were palatally displaced due to lack of space; the upper right lateral incisor was rotated 180 degrees; the upper left lateral incisor was rotated 90 degrees; and there was a severe upper crowding (14 mm). The lower arch was U-shaped with slightly constricted intercanine distance (20 mm) and a mild generalized crowding (4 mm) (Figure 2).

**Figure 3:**
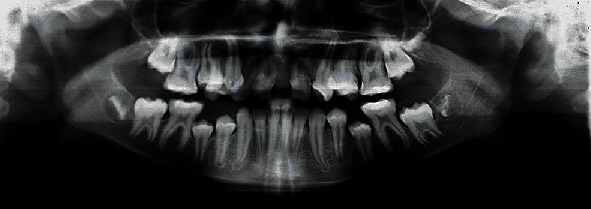
Pretreatment pantomogram X-ray.

Panoramic radiograph showed normal findings except for unerupted lower premolars, unerupted all permanent second molars, and dilacerated roots of many teeth: #11, 15, 16, 21, 25, and 26 (Figure 3).

**Figure 4:**
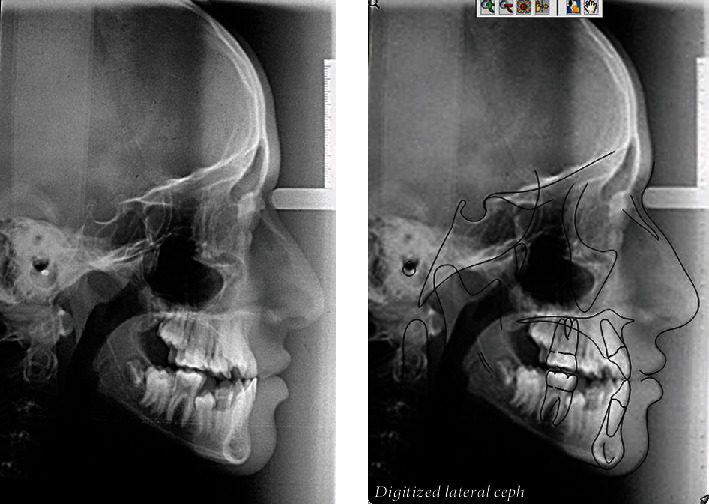
Pretreatment cephalometric radiograph and its tracing.

The cephalometric analysis showed a skeletal class III relationship ANB (-3°), due to retrognathic maxilla (SNA = 77°) and an average mandible (SNB = 80). The class III pattern was also confirmed by Wits appraisal (-4 mm). A lower anterior facial height was within normal limits, and the maxillary-mandibular plane angle value was also average (22°). The upper incisors were normally inclined, and the lower incisors were retroclined and retruded. The interincisal angle was found to be increased (142). Lips were retruded; the lower lip was -6 mm to the Ricketts E-line [10], the upper lip was -4 mm to the Ricketts E-line, and the nasolabial angle was increased (119°) (Figure 4, Table 1).

Cervical vertebra maturation (CVM) showed that the peak of mandibular growth would be expected to be 1 year after this stage (CS3) [11].

### 2.1. Treatment Objectives

The treatment performed in the present case was a nonextraction treatment, which considered improving the retruded lips and reestablishing the proper arch dimensions anteroposteriorly and transversely, despite the presence of 14 mm of crowding and multiple severely displaced teeth. The main treatment objectives were (1) to improve the patient's facial and dental aesthetics, (2) to expand upper and lower arches to their original dimensions, (3) to redistribute spaces to gain spaces for teeth alignment, (4) to achieve class I molar and canine relationship with an optimum overbite and overjet, (5) to achieve a mutually protected occlusal scheme with maximum intercuspation, and (6) to accept the mild skeletal discrepancy.

### 2.2. Treatment Alternative

Rapid palatal expansion and face mask therapy were used to correct the class III discrepancy. This was excluded because the prognosis of skeletal protraction is questionable at this age [12], and there is difficulty in inserting RPE palatally in the presence of the severely displaced teeth. Extraction therapy was also suggested as a treatment option, which was less acceptable to avoid the risk of dishing the lips.

### 2.3. Treatment Planning and Progress

The treatment started with the bonding of the upper and lower arches using 0.018 Roth brackets (Gemini, 3M Unitek, Dental Products, Monrovia, CA), excluding the upper lateral incisors and upper second premolars in the upper arch. A trihelix expander was soldered to the molar bands and used to expand, derotate, and distalize the upper first molars. Bonding of the lower arch was then done, and a lip bumper appliance was used in the lower arch to regain space and to allow smooth eruption of the lower second premolars. After 2 months of active treatment, the bonding of lower arch was done, excluding the unerupted lower second premolars, and unerupted lower right first premolar, in addition to the lower right canine (as the bracket of the canine can interfere with the planned lip bumper appliance).

**Figure 5:**
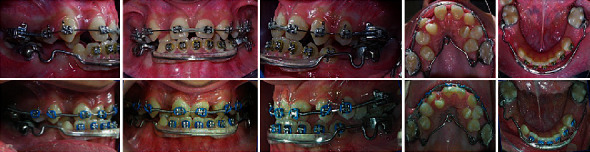
Midtreatment photographs.

A second phase treatment was started after the eruption of the full permanent dentition, excluding the third molars. Arch coordination and finishing were done for 6 months (Figure 5). The retention plan consisted of full-time wear of an upper wraparound retainer, upper and lower anterior fixed lingual retainers, upper frenectomy, and fibrotomy for rotated teeth.

### 2.4. Treatment Results

**Figure 6:**
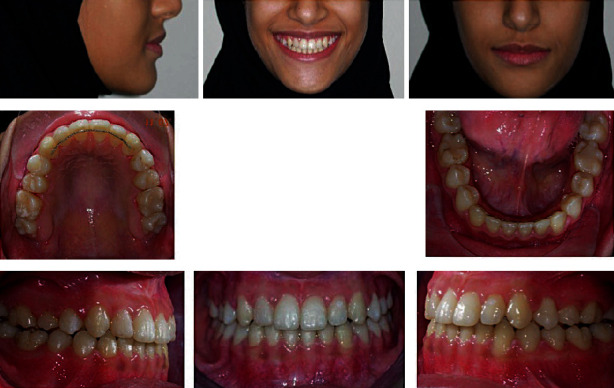
Posttreatment extraoral and intraoral photographs.

Treatment was accomplished in 24 months. The crowding was resolved, the severely displaced teeth were aligned bodily, and normal overbite and overjet were achieved with class I canine and molar relationships (Figure 6). A three-year follow-up indicated stable results in all parameters.

**Figure 7:**
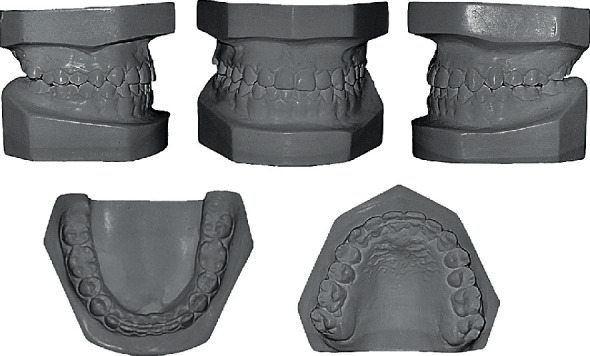
Posttreatment study models.

There was a 4 mm increase in the intermolar distance in the upper arch and 2 mm in the lower arch. The intercanine distance was maintained except for a 1 mm increase in the upper arch (Figure 7).

Regarding aesthetic outcomes and limitations, the smile was greatly enhanced by reducing the buccal corridor with the expansion appliance and producing a maxillary incisal plane parallel to the lower lip by proper leveling and alignment of the teeth.

A noticeable improvement of the facial profile was also produced by advancing the upper lip into a more favorable position in relation to the lower lip.

**Figure 8:**
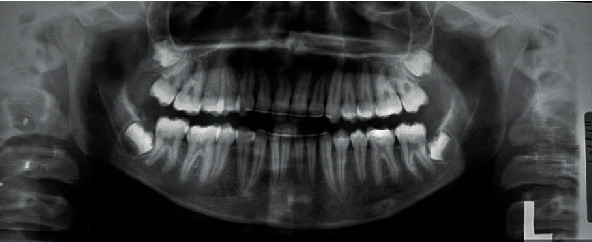
Posttreatment pantomogram X-ray.

**Figure 9:**
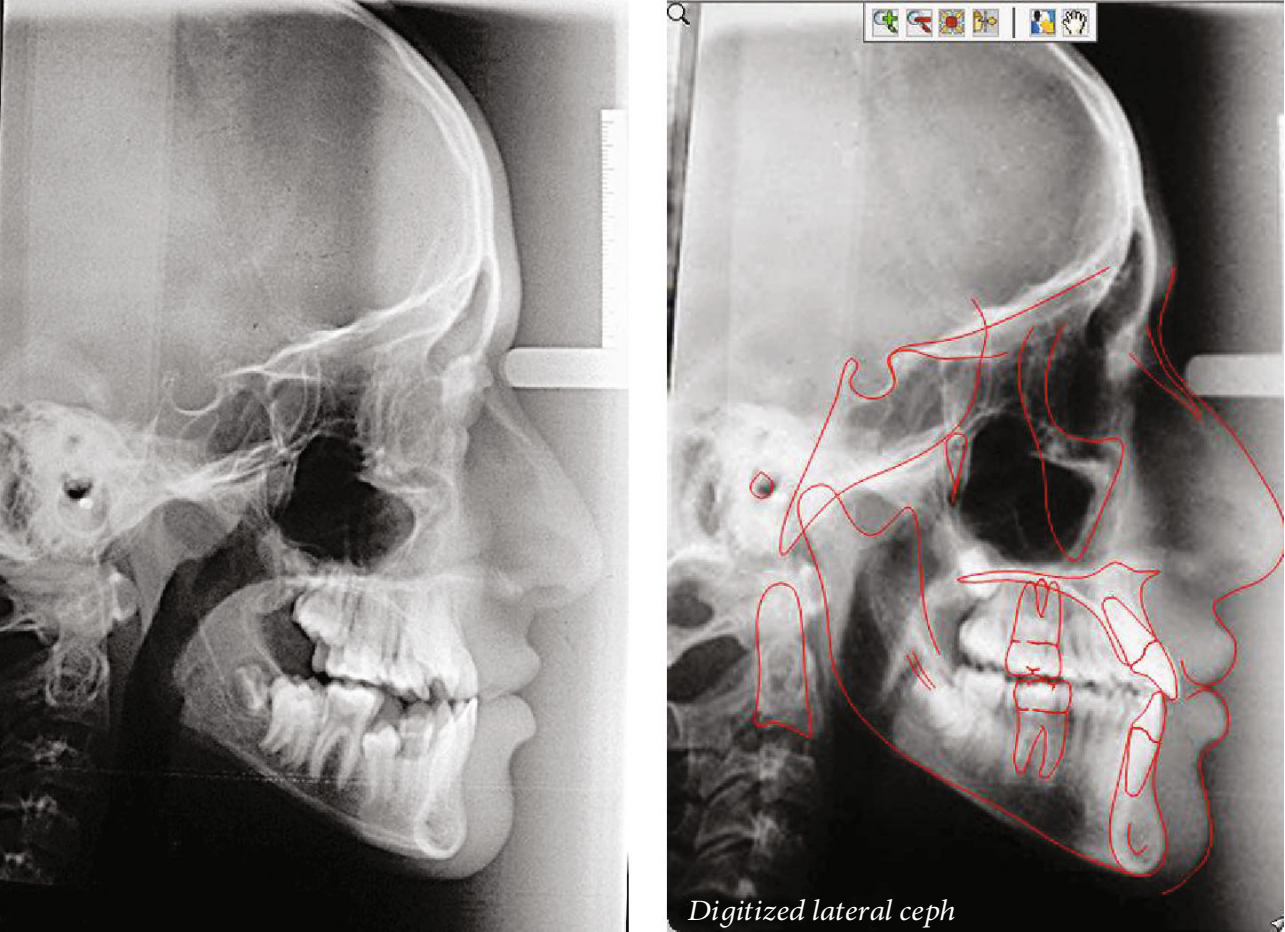
Posttreatment cephalometric radiograph and its tracing.

**Figure 10:**
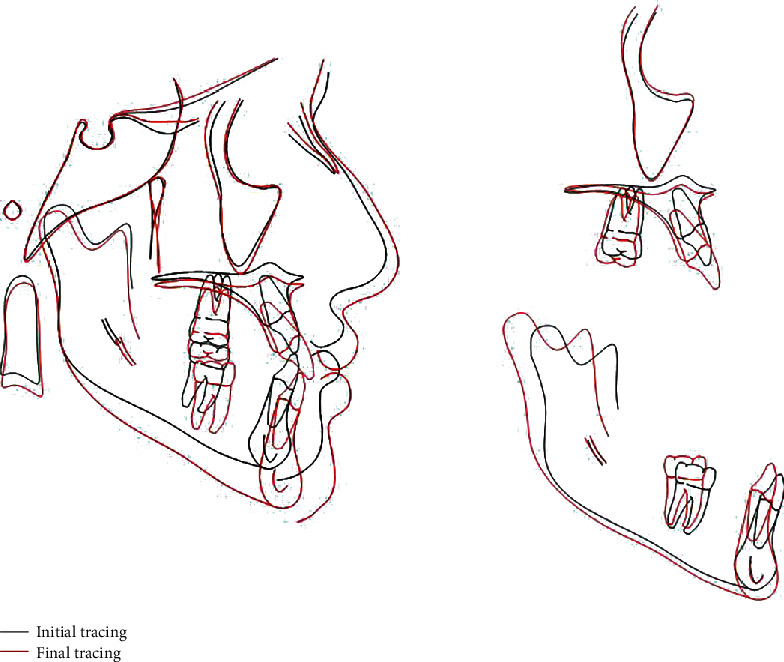
Cephalometric superimposition.

A panoramic X-ray and cephalometric analysis showed insignificant changes facially, and good lip balance and harmony were achieved due to the proclination of the upper and lower incisors (Figures 8–10 and Table 2).

### 2.5. Retention

**Figure 11:**
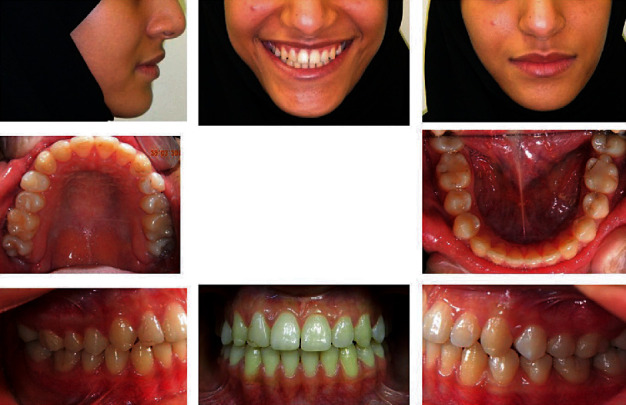
Postretention extraoral and intraoral photographs.

The 3-year postretention records showed stable results, although the patient was having only the upper and lower fixed retainers without wearing any removable retainer at this stage (Figure 11).

## 3. Discussion

This case report demonstrates the importance of considering the lip prominence, the patient age, the dental arch configuration, and the application of basic biomechanics during the treatment planning of cases having crowding. Although the patient had severe crowding (14 mm), it was relieved via expansion and the application of the reciprocal force system to distalize the upper first molars against incisor proclination to the desired position. Also, spaces were redistributed between opening spaces for the alignment of premolars and closing of the large midline diastema.

Treatment of any crowded dental arch usually requires space gain. This space can be gained in two ways, either through extraction or through nonextraction. In growing patients, nonextraction methods to gain space, such as expansion, distalization, and proclination, are usually preferred. This is to make use of the active period of growth to enhance the growth of the basal bone and dentoalveolar complex into a more favorable direction. Also, distalization in the early stage is much easier and has a more predictable outcome [13, 14].

The nonextraction approach was selected in the present case to improve the patient's profile as it protruded the lips, which was suitable for her age. It also demonstrated the efficiency of combining a trihelix appliance and a fixed orthodontic appliance with push coils to reciprocally move molars and incisors apart to create adequate space for such severe crowding. The trihelix is a modified quadhelix fixed expander made of 0.028 stainless steel wire with three helices instead of four helices, which makes it less bulky than the quadhelix and suitable for cases with severe palatal constriction, where there is no room to fit a palatal expander. The present case presents the best example for such an indication for the use of a trihelix expander since there was no room in the palatal space for a quadhelix due to the presence of the severely palatally displaced premolars. At the same time, the trihelix was indicated to derotate the upper first molars, as seen in this case, where it helped to provide space for the malaligned premolars.

The expansion and alignment of the upper arch alleviated the present functional shift of the mandible and caused a spontaneous correction of the midline. The intermolar width was expanded by 4 mm, while the intercanine width was not changed. A dilaceration was introduced to the patient as a possible risk factor for root resorption during the treatment.

The fixed orthodontic appliance allowed for perfect leveling and aligning of the teeth. The upper lateral incisors were severely rotated, which could have taken a long time to derotate them and exposed them to root resorption. In this case, it was possible to derotate the upper right lateral incisor to its original orientation. However, the attempt to align #12 was not accomplished; the rotation was accepted as it could have needed a longer time to align it within the arch. Also, the excessive derotation forces and lengthy time might increase the risk of root resorption [15]. Esthetically, the tooth was restored by composite veneer, which made it esthetically pleasant. Labial root torque was performed successfully on both upper lateral incisors for better stability. The diastema was properly addressed during treatment and retention. It was closed via bodily movement of incisors, stabilized by performing frenectomy, and then retained by a fixed lingual retainer.

The lip bumper is known to alter the equilibrium forces on the lower dental arch and allow the physiological widening of the arches, as well as distal tipping of the lower first molars [8, 9]. In the lower arch, a lower lip bumper was efficient to tip the lower molars distally and create extra space for relieving lower crowding. The lip bumper was constructed to be low enough to allow the lip to rest on the teeth and decrease the amount of flaring of the lower anterior teeth. Also, lower brackets were bonded during the lip bumper therapy, which helped to decrease the flaring effect of the appliance on the anterior teeth. Based on cephalometric superimposition, the lip bumper tipped lower molars distally 1 mm; it also widened the lower arch by 1.5 to 3 mm at canines and molars, respectively. A slight overjet (1.5 mm) existed between the upper central and lower incisors. This was due to the interference between the rotated buccal surface of tooth #22 with the lower incisors. Although some occlusal adjustment was made during the composite preparation of tooth #12, the more palatal reduction was needed; however, this was not done to avoid excessive tooth hypersensitivity, which was questioned by the patient.

Follow-up radiographs have shown no visible resorption of any of the dilacerated roots of the upper centrals and laterals. In addition, there was root approximation between upper premolars, which was verified by periapical radiographs, which have shown the presence of adequate bone between roots. No attempt was made to correct the root angulations as the occlusion, and the crowns were in the proper position.

Using the Björk and Skieller method of superimposition [16], the overall superimposition showed a normal growth pattern. Maxillary superimposition showed that maxillary incisors moved forward, downward, and proclined relative to their previous position. Maxillary molars moved backward. Mandibular superimposition showed that lower incisors were proclined from their original position, and lower molars were uprighted 1 mm backward.

Fixed and removable retainers were used after debonding. The initial anterior crowding, upper diastema, and rotations all represented a strong indication for fixed retention, which was done in the present case. Overlay removable retainers, of the wraparound type, were used in the upper and lower arches. This design was preferred because it does not interfere with the occlusion and allows the teeth to settle. In addition, it is assumed to maintain the achieved expansion in the upper arch and helps in closing the band spaces. The patient was willing to wear the retainers as directed. Follow-up records after 3 years show great stability, and the patient was advised to keep the fixed retainers. This indicates the importance of the methods of retention used in the present study.

## 4. Conclusion

The decision of extraction versus expansion is critical, even in cases with severe crowding. The lip position, the arch configuration, and the age are important factors to consider when planning treatments for such cases.

## Figures and Tables

**Table 1:** Pretreatment cephalometric analysis.

Variable	Pretreatment	Normal
SNA	77°	82° ± 3
SNB	80°	79° ± 3
ANB	-3°	3° ± 1
SN to maxillary plane	8°	8° ± 3
SN to mandibular plane	30°	32°
Wits appraisal	-5 mm	0 mm
Upper incisor to maxillary plane angle	110°	108° ± 5
Lower incisor to maxillary plane angle	85°	92° ± 5
U6–PTV	11.9 mm	—
L6–PTV	13.2 mm	—
Interincisal angle	142°	133° ± 10
Maxillary-mandibular plane angle	22°	27° ± 5
Upper anterior face height	51	—
Lower anterior face height	63	—
Face height ratio	55%	55%
Lower incisor to APo line	4 mm	0–2 mm
Lower lip to Ricketts plane	-6 mm	-2 mm
Upper lip to Ricketts line	-4 mm	-4 mm
Nasolabial angle	119°	102° ± 8
Mand length (co-Pog)	113 mm	117 mm
Max length (co-A)	86 mm	92 mm
Max/mand differential	27 mm	27 mm

**Table 2:** Posttreatment cephalometric analysis.

Variable	Pretreatment	Posttreatment
SNA	77°	78°
SNB	80°	80°
ANB	-3°	-2°
SN to maxillary plane	8°	10°
SN to mandibular plane	30°	32°
Wits appraisal	-5 mm	-3 mm
Upper incisor to maxillary plane angle	110°	117°
Lower incisor to maxillary plane angle	85°	93°
Interincisal angle	142°	127°
U6–PTV	11.9 mm	10.3 mm
L6–PTV	13.2 mm	12.4 mm
Maxillary-mandibular plane angle	22°	20°
Upper anterior face height	51 mm	54 mm
Lower anterior face height	63 mm	64 mm
Face height ratio	55%	54%
Lower incisor to APo line	4 mm	3 mm
Lower lip to Ricketts plane	-4 mm	-2 mm
Upper lip to Ricketts line	-6 mm	-3 mm
Nasolabial angle	119°	100°
Mand length (co-Pog)	113 mm	115 mm
Max length (co-A)	86 mm	87 mm
Max/mand differential	27 mm	28 mm
